# An economic analysis of usual care and acupuncture collaborative treatment on chronic low back pain: A Markov model decision analysis

**DOI:** 10.1186/1472-6882-10-74

**Published:** 2010-11-25

**Authors:** Namkwen Kim, Bongmin Yang, Taejin Lee, Soonman Kwon

**Affiliations:** 1 Seoul National University, Graduate School of Public Health, Seoul, South Korea; 2 Oriental Medical College of Wonkwang University, Iksan, South Korea

## Abstract

**Background:**

The collaborative treatment of acupuncture in addition to routine care as an approach for the management of low back pain (LBP) is receiving increasing recognition from both public and professional arenas. In 2010, the Ministry of Health, Welfare and Family Affairs (MOHW) of South Korea approved the practice of doctors and Oriental medical doctors (acupuncture qualified) working together in the same facility and offering collaborative treatment at the same time for the same disease. However, there is little more than anecdotal evidence on the health and economic implications of this current practice. Therefore, the objective of this study is to examine the effectiveness and costs of acupuncture in addition to routine care in the treatment of chronic LBP patients in South Korea.

**Methods:**

The Markov model was developed to synthesise evidence on both costs and outcomes for patients with chronic LBP. We conducted the base case analysis, univariate and probabilistic sensitivity analyses, and also performed the value of information analysis for future researches. Model parameters were sourced from systematic review of both alternatives, simple bibliographic reviews of relevant articles published in English or Korean, and statistical analyses of the 2005 and 2007 Korean National Health and Nutrition Survey (KNHNS) data. The analyses were based on the societal perspective over a five year time horizon using a 5% discount rate.

**Results:**

In the base case, collaborative treatment resulted in better outcomes, but at a relatively high cost. Overall, the incremental cost-effectiveness ratio of a collaborative practice was 3,421,394 KRW (Korean rate Won) per QALY (Quality adjusted life year) (2,895.80 USD per QALY). Univariate sensitivity analysis of indirect non-medical costs did not affect the preference order of the strategies. Probabilistic sensitivity analysis revealed that if the threshold was over 3,260,000 KRW per QALY (2,759.20 USD per QALY), the probability for cost-effectiveness of a collaborative practice would exceed 50%. At 20,000,000 KRW per QALY, which is recommended using per capita gross domestic product (GDP) as the threshold, the probability would be 72.3%.

**Conclusions:**

On the basis of our findings, acupuncture collaborative therapy for patients with chronic LBP may be cost-effective if the usual threshold is applied. Further empirical studies are required to overcome the limitations of uncertainties and improve the precision of the results.

## Background

Numerous studies in various countries attest to the high frequency of low back pain (LBP). Approximately 70-85% of all people have back pain at some point in their lifetime, and the annual prevalence ranges from 15% to 45%, with point prevalence averaging 30%. Nearly 80-90% of patients with back pain recover quickly within 12 weeks, but recovery after 12 weeks is slow and uncertain[1]. Chronic LBP is defined as LBP that persists or recurs over 12 weeks [1,2]. A 2007 Korean National Health and Nutrition Survey (KNHNS) data analysis showed that over 6.5% of adults experience chronic LBP [3]. Chou et al. [4] stated that there has been little consensus on the management of LBP, and the development of clinical practice guidelines based on available evidence is necessary.

In some countries, the addition of acupuncture treatment, compared to usual care alone, has proved to be cost-effective [5,6]. However, some parameters, such as natural mortality rates, medical costs and national threshold, which are necessary in the analyses, differ from country to country. Therefore, cost-effectiveness results may also vary by medical institution and conditions; an economic evaluation should be conducted for each situation [7].

Decision analytic modelling is a systematic approach to decision making under uncertainty that is widely used in economic evaluations of pharmaceuticals and other health care technologies [7]. Despite the concerns about the methodologies of economic evaluation [8], decision analytic modelling is used to synthesise the best available data and conduct economic evaluations, especially when no optimal cost-effective analytic outcome from clinical trials has yet been established [9].

Recently, the Korean Ministry of Health, Welfare and Family Affairs (MOHW) launched several laws concerning medical provider employment and collaboration. Under these laws, doctors and Oriental medical doctors (acupuncture qualified) can work together in the same facility and offer collaborative treatment at the same time for the same disease [10]. Until 2009 in South Korea, these types of collaborations were indirectly regulated by the limited reimbursing regulation of Health Insurance Review and Assessment service (HIRA). Therefore, there is a possible increase in chronic LBP cases that are treated with collaborative treatment in addition to the usual practice.

The purpose of this study is to examine the cost-effectiveness of usual care and acupuncture collaboration as compared to usual care alone, and to provide information about the level of improvement required to substantially alter the cost-effectiveness of the therapeutic decision in South Korea. We also conducted a value of information analysis, using the net monetary benefit and population expected value of perfect information (EVPI), to provide a rational background for future research investments.

## Methods

### Treatment regimen

#### Usual Care

The American College of Physicians and the American Pain Society (ACP&APS) promoted clinical practice guidelines for chronic LBP, that achieve at least grade B results (medium benefit and/or no harm) according to prior research [11]. The interventions listed in the guideline include self-care (remain active, hand out books and apply superficial heat), pharmacological therapies (acetaminophen, NSAIDs, antidepressants, benzodiazepines and opioids) and non-pharmacologic therapies (spinal manipulation, exercise therapy, massage, acupuncture, yoga, cognitive-behavioural therapy, progressive relaxation and intensive interdisciplinary rehabilitation). Among these interventions, orthopaedic and rehabilitation specialists in a general hospital were asked for common prescriptions that have been established as generalised 'usual care' lists used in South Korea. These interventions included NSAIDs, heat therapy, electrotherapy and lumbar traction (Table 1).

**Table 1:** Definitions of procedures and medical costs (KRW in 2009) 24

Procedure	First visit		Regular visit for diagnosis and treatment		Simple regular visit for treatment	
	
	Treatment	Cost	Treatment	Cost	Treatment	Cost
Usual Care	First medical examination	14,730	Recursive medical examination	11,080	Hospital management fee	3,110
	
	Diagnostic testing	18,648	Diagnostic testing	7,510	Physical treatment	7,439
	
	Pharmacy cost	7,510	Pharmacy cost	5,650		
	
	Drug cost	5,650	Drug cost	7,439		
	
	Physical therapy	7,439	Physical therapy	31,685		

Frequency	1 time	53,983	6 times	31,685	3 times	10,549

Direct medical costs for usual care during 3 months in the chronic LBP state.					275,740	

Usual Care and Acupuncture Collaborative Treatment	First medical examination	14,730	Recursive medical examination	11,080	Hospital management fee	3,110
	
	First oriental medical examination	9,980	Recursive oriental medical examination	6,300	Physical treatment	6,300
	
	Collaborative examination	4,120	Collaborative examination	4,210	General acupuncture	7,439
	
	Pharmacy cost	7,510	Pharmacy cost	7,510	Special spine acupuncture	4,212
	
	Drug cost	5,650	Drug cost	5,650		3,816
	
	Physical therapy	7,439	Physical therapy	7,439		
	
	General acupuncture	4,212	General acupuncture	4,212		
	
	Special spine acupuncture	3,816	Special spine acupuncture	3,816		

Frequency	1 time	57,463	6 times	50,223	3 times	24,877

Direct medical costs for collaboration during 3 months in the chronic LBP state.					433,434	

Pharmacy cost: pharmacy management (14 days) + prescription cost + medication teaching cost + pharmaceutical cost Physical treatment: Hot pack, TENS, traction

#### Usual care and acupuncture collaboration

The definition of collaboration in this study is that the usual care provided by medical doctors, and the acupuncture treatment provided by licensed Oriental medicine doctors, are collaboratively offered to patients at the same time in the same hospital. Because clinical studies regarding the usual care and acupuncture collaborative treatment of chronic LBP in South Korea have not been conducted, we systematically reviewed the papers and derived the effectiveness results (Appendix 1-2). We also found similarities within the acupuncture treatment protocols from the systematic review results (Table 2), and assumed that the effectiveness of acupuncture by Oriental medicine doctors would be the same based on Cherkin et al.'s result [12].

**Table 2:** Acupuncture protocols for chronic low back pain in the papers

Paper and study type	Nation	Type of acupuncture	Theory and rationale	Treatment area and acupuncture points	Treatment sessions	Treatment duration (min)
Carlsson (2001) RCT [30]	Sweden	Disposable, stainless steel, diameter between 0.30 mm and 0.32 mm, length between 30 mm and 70 mm	Not-mentioned	Lower back lower limbs, forearms or hands (Bladder and large intestine acupuncture points)	8 session per 8 weeks	20 minutes

Leibing (2002) RCT [34]	Germany	Wrapped one-way stainless steel, sterilised needle diameter 0.30 mm length40 mm	Traditional Chinese medicine	Body and ear (Governor Vessel, bladder, gall bladder, spleen acupuncture points)	20 session per 12 weeks	30 minutes

Kerr (2003) RCT [32]	Northern Ireland	Not-mentioned	Not-mentioned	Bladder, gall bladder, kidney meridian and governor vessel acupuncture points	6 session per 6 weeks	30 minutes

Meng (2003) [35]	USA	Disposable, sterile 30-gauge needles	Traditional Chinese medicine	Urinary bladder meridian	10 session per 5 weeks	Not-mentioned

Thomas (2006) Pragmatic RCT [25]	UK	Sterilised, disposable needle, needle length and diameter were not predefined	Not-mentioned	Bladder, gall bladder meridian acupuncture points	10 session per 3 months	Not-mentioned

Brinkhaus (2006) RCT [29]	Germany	Sterile, disposable needle length 50 mm	Not-mentioned	bladder, governor Vessel, small intestine, bladder, kidney, gall bladder meridian acupuncture points	12 session per 8 weeks	30 minutes

Kwon RCT (2007) [33]	S. Korea	Disposable stainless needle	Donguibogam	Gall bladder, small intestine, bladder meridian acupuncture points	12 session per 4 weeks	20 minutes

HIRA (2009) [30]	S. Korea	40 mm length, 0.30 mm width, stainless steel metal	Acupuncture (Textbook)	Bladder, Du mai channel, small intestine, gallbladder, channel etc.	Not-mentioned	20 minutes

### Model Structure

We constructed a Markov model and conducted the analyses based on the references using available software, Microsoft Excel 2007 [13]. Treatment regimens were compared as defined above and we assumed that there were 10 treatment sessions per cycle.

**Figure 1:**
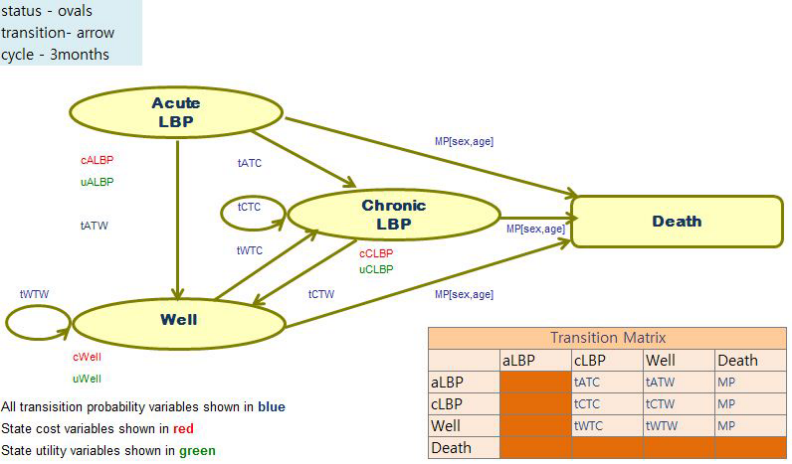
**Markov model of chronic low back pain**.

Markov states were acute LBP, chronic LBP, Well, and Death states (Figure 1). The cohort of patients was assumed to be 60-year-old females, who recorded the highest prevalence rate of suffering from their first experience of acute LBP according to the 2007 KNHNS results. Individuals who experienced LBP for the first time for no more than three months were included in the initial acute LBP state.

Patients, for whom the pain lasted over three months in the initial acute LBP state, were transitioned to chronic LBP. Those patients who recovered from acute LBP and chronic LBP moved to the Well state. If the pain reoccurred in the Well state, they were retransferred to the chronic LBP state. Cases in all states could be transferred to the Death state based on age- and sex-specific all-cause mortality rates derived from South Korean life tables [14]. The effectiveness of acupuncture for acute LBP has not yet been proven [15-17], therefore we assumed that the patients of both treatment groups in the acute LBP state were equally treated by usual care, and were excluded from the analysis.

The analyses were based on a societal perspective over a five-year time horizon using a 5% discount rate. We defined the time horizon according to the revision schedule of National Health Insurance Medical Costs and the discount rate based on the reference case recommendation of the Panel on Cost-Effectiveness in Health and Medicine and the Guidelines of Economic Evaluation of Medical Supplies in South Korea [18].

All monetary costs were converted to 2009 Korean rate Won (KRW) using the South Korean Consumer Price Index [19]; exchange rates used in the analysis were in accordance with the 2009 Korean Exchange Rates (1 USD = 1,181.50 KRW) [20]. The Markov cycle length was three months and the time horizon was twenty cycles. Effectiveness obtained from both alternatives was calculated using quality adjusted life year (QALY) to account for changes in quality of life (QOL). Key assumptions for construction of the model are listed under each component below and all source data are openly available in the mentioned databases.

### Model Estimation

#### Transition Probabilities

Probabilities from the initial state to the chronic LBP and Well state were estimated from a perspective cohort study by Grotle et al., in which the first-time patients consulted primary care due to an episode of acute LBP. Of this group, 24% still experienced LBP after three months [21]. We assumed that the recurrence of LBP in the Well state is considered as chronic LBP. The recurrence rate was derived from the Cassidy et al. study [22]. The recurrence rate of 6 months in the study is converted to the transition probability of 3 months using the following formulae [23]:

Probability=1−exp(−Rate×time), Rate=−[ln(1−Probability)]/time

**Figure 2:**
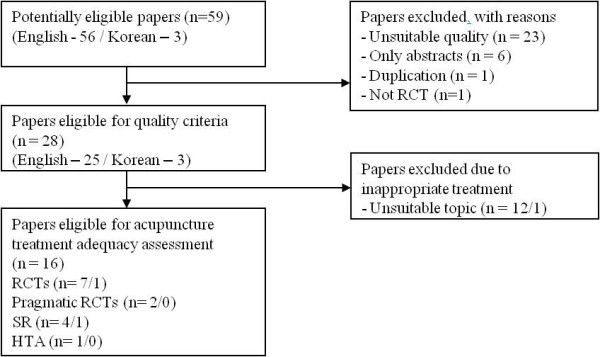
**Systematic review flow diagram**.

**Figure 3:**
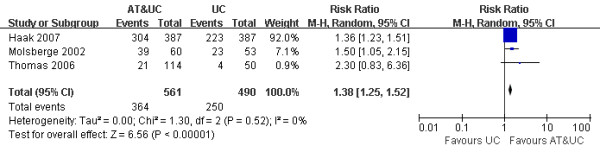
**Meta-analysis of the risk ratio (transition probability)**.

The different transition probabilities of both alternatives from chronic LBP to Well state were obtained from a systematic review and meta-analysis results. Considering the development of medical technologies and supplies, we defined the review period as 10 years from January 1999 to January 2009. The flow diagram of the systematic review is described in Figure 2, and the meta-analysis results are shown in Figure 3. Because the studies included in the meta-analysis are from different countries, we used the random effect model to overcome the heterogeneity. From the analysis, we achieved the difference of transition probabilities between two alternatives as a risk ratio of 1.40. All transition probabilities are described in Table 3 and the transition matrix is defined in Table 4.

**Table 3:** Parameter values and distributions examined in sensitivity analyses

Variable groups	Name (Citation)	'Live' value	Probabilistic	Deterministic	Standard error	Distribution	alpha	beta
Transition probability(Tp) variables	tATC [21]	0.24	0.04	0.24	28.773	Gamma/Normal	29	91
	
	tATW [21]	0.76	0.13	0.76	86.328	Gamma/Normal	91	29
	
	tWTC [22]	0.16	0.04	0.16	28.410	Gamma/Normal	26	137
	
	tWTW [22]	0.84	0.21	0.84	159.883	Gamma/Normal	137	26
	tCTW [31]	0.35	0.32	0.35	0.024	Beta	135	252

Resource cost parameters	cUC [41]	507,776 KRW	543,520 KRW	494,071 KRW	507,776 KRW	Gamma	24.69	20566.20
	
	cACUC [41]	730,329KRW	706,676 KRW	682,759 KRW	730,329 KRW	Gamma	19.54	37372.18

Utility of Markov states per cycles	uALBP [3]	0.85	0.95	0.85	0.15	Beta	3.97	0.70
	
	uCLBPUC [5]	0.62	0.68	0.62	0.10	Beta	13.99	8.57
	
	uCLBPACUC [5]	0.65	0.70	0.65	0.10	Beta	14.14	7.61
	
	uWell [3]	0.96	0.93	0.96	0.04	Beta	22.08	0.92

Recovery rate of chronic LBP	tRR (Fig. 2)	1.40	1.31	1.40		Log normal	0.34 (Ln-mean)	0.05 (Ln-SE)

TP parameters

tATC: TP acute LBP to chronic LBP state, tATW: TP acute LBP to Well state, tWTC: TP Well to chronic LBP state, tWTW: TP Well to Well state, tCTW: TP chronic LBP to Well state, Resource costs parameters

cUC: costs used in chronic LBP state treated by usual care, cACUC: costs used in chronic LBP state treated by collaboration Utility parameters

uALBP: QOL of acute LBP state, uCLBPUC: QOL of chronic LBP state treated by usual care, uCLBPACUC: QOL of chronic LBP state treated by collaboration, uWell: QOL of Well state Recovery rate

tRR: risk ratio of TP from chronic LBP to the Well state when treated by the collaboration compared with the usual care

**Table 4:** Transition matrix (Usual care)

Transition Matrix				
	**aLBP**	**cLBP**	**Well**	**Death**

**aLBP**		tATC = (1-mr) × 0.24	tATW = (1-mr) × 0.76	Mp

**cLBP**		tCTC = (1-mr-tCTW)	tCTW = (1-mr) × 0.35	Mp

**Well**		tWTC = (1-mr) × 0.16	tWTW = (1-mr) × 0.84	Mp

**Death**				

1) Mp (mortality probability) : South Korea Life Table

2) tATC, tATW: Grotle et al. (2005) [21]

3) tWTC, tWTW: Cassidy et al. (2007) [22]

4) tCTW: Haake et al. (2007) [31]

#### Utilities

Estimate of acute LBP and Well state utilities were derived from a subgroup analysis of 2007 KNHNS data. No domestic research results estimating the utilities of chronic LBP were identified, thus we estimated separate utilities for the usual care and acupuncture collaboration groups from the Witt et al. study [5]. All states' QOLs are listed in Table 3.

#### Costs

Direct costs of both alternatives for 1 cycle (3 months) in chronic LBP were obtained from the South Korean National Health Insurance Reimbursement for standard medical procedures [24], and frequencies were derived from pragmatic trial results [5,25]. Direct non-medical costs, such as traffic expenses, waiting times, and treatment times obtained from the 2005 KNHNS data analysis were included in the cost simulation [26]. All medical costs for both alternatives are listed in Table 1. Direct non-medical costs and indirect non-medical costs were obtained using sources and formulae as described in Table 5. The costs of the acute LBP and Well states were excluded, based on the assumptions firstly that the treatment for acute LBP would be the same, and secondly that no treatment would be required in the Well state.

**Table 5:** Costs of 3 months in the chronic low back pain state (KRW in 2009)

Strategy	Cost (KRW)
Usual care	
Direct medical costs	275,740
Direct non-medical costs	232,036
Indirect non-medical costs	239,142

Total cost for usual care	746,918

Collaborative treatment	
Direct medical costs	433,432
Direct non-medical costs	296,897
Indirect non-medical costs	239,142

Total cost for collaborative treatment	969,471

Direct non-medical costs [26]: Hospital visiting time (15.64 min) + Treatment time (Usual care - 60 min, Collaboration -90 min) + Traffic expenses (6,850 KRW)

Indirect non-medical costs [41]: Indirect cost of both alternatives = Average wage per day (65,333 KRW) × Proportion of economically active person (0.614)× Percentage of employment (0.586) × Number of treatment sessions (10)

### Statistics and Analyses

Deterministic analyses, univariate and probabilistic sensitivity analyses were performed. In the deterministic analysis, we entered the mean values of the parameters and calculated the incremental cost-effectiveness ratio (ICER) of both alternatives. Recently, when using QALYs as the denominator, whether to include the indirect non-medical costs in the analysis has become debatable [18,27]. Therefore, in the base case analysis, we excluded the indirect non-medical costs from the analysis, and then examined whether including these costs could alter the cost-effectiveness result using univariate sensitivity analysis.

In the probabilistic sensitivity analyses, all parameters were varied simultaneously over their listed range, with 10,000 recalculations of net benefits using random draws from their distributions, as shown in Table 3.

In addition, we calculated the population EVPI using probabilistic sensitivity analysis results over a 5-year time horizon, assuming 57,400 cases per year, calculated from the 2007 KNHNS data. The EVPI estimates the value of eliminating uncertainty in all parameters and is calculated by subtracting the expected net benefit of adopting an intervention based on current information from the expected net benefit with perfect information. The EVPI is the maximum that decision makers should be willing to pay to resolve uncertainty about the adoption of an intervention. In this context, when launching certain research projects, the EVPI calculation determines whether it is worthwhile to resolve the uncertainty.

Following Briggs et al.'s book [23], we used Microsoft Office Excel 2007 to develop our model and conduct the analyses. The systematic review and meta-analysis were conducted using Review Manager 5. To obtain the mean values and distributions of parameters, simple descriptive statistics, Student's t-test, and ANOVA were executed using the Stata SE 10 program.

## Results

### Model calibration and validation

Before analysing the model, we tested the validity of the Markov model to determine whether the results produced by the 'usual care' option reflect the real incidence of chronic LBP. Because no comparable domestic clinical cohort study result was found, we compared the Canadian recurrence rates of 20% in 1 year and 36% over 3 years [1]. The mean number of cases of chronic LBP during the simulation periods revealed 2,982 cases (95% confidence interval: 2,911-3,052) per 10,000 initial state patients, and the point estimate of model outputs after 5 years was 29.71% - close to the Canadian recurrence rate of 20% in 1 year and 36% over 3 years.

### Deterministic analysis

**Figure 4:**
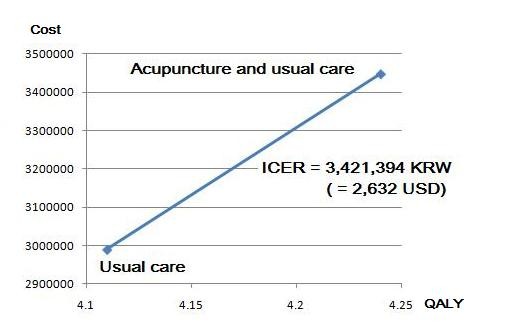
**ICER between two alternatives (deterministic result)**.

In the base case, for the 10,000 60-year-old female cohorts with acute LBP, the usual care resulted in a discounted gain of 4.11 QALYs and cost of 2,988,203 KRW per one person over 5 years. Collaborative treatment resulted in a discounted gain of 4.24 QALYs and cost of 3,447,840 KRW. The ICER, which was derived from both results, was 3,421,394 KRW per QALY (2,895.80 USD per QALY) (Table 6). Because no absolute cost-effectiveness criterion exists, we used WHO recommended guidelines, which suggest using per capita GDP (20,000,000 KRW) as the threshold for each country [28]. Therefore, the ICERs for collaborative treatment versus usual care are significantly less than the threshold. The cost-effectiveness plane is displayed in Figure 4.

**Table 6:** Deterministic analysis results (KRW in 2009)

Strategy	Cost (KRW)	Incremental cost	Effectiveness (QALY)	Incremental effectiveness	Incremental C/E ratio
Usual care	2,988,203	-	4.11	-	-
Collaborative treatment	3,447,840	459,637	4.24	0.13	3,421,394

### Sensitivity analysis

#### Univariate sensitivity analysis

We performed univariate sensitivity analysis of indirect non-medical costs in the model. This analysis was conducted to determine if adding the indirect costs to the value of total costs would change the results of the analysis. Productivity loss of the patients was calculated using the following formula:

Average wage (1day)×Proportion of economically active people×Percentage of employment.

The total indirect non-medical costs of both alternatives were assumed as 239,142 KRW per 3 months. Including indirect non-medical costs in the analysis did not changes the preference order of the strategies, as described in Table 7.

**Table 7:** Univariate sensitivity analysis of indirect cost (KRW in 2009)

Variable	Indirect cost per 1 patient (KRW)	Total cost per 1 cycle (KRW)	Δ Cost per time period	Δ QALY per time period	Incremental C/E ratio
Base case					
Usual care	0	507,776			
Collaborative treatment	0	730,329	3,421,394	0.13	3,421,394

Inclusion of indirect cost					
Usual care	239,142	746,918			
Collaborative treatment	239,142	969,471	181,290	0.13	1,349,463

Δ Cost = Total cost of acupuncture collaboration - Total cost of usual care

Δ QALY = QALY gained with the acupuncture collaboration - QALY gained with the usual care

#### Probabilistic sensitivity analysis

**Figure 5:**
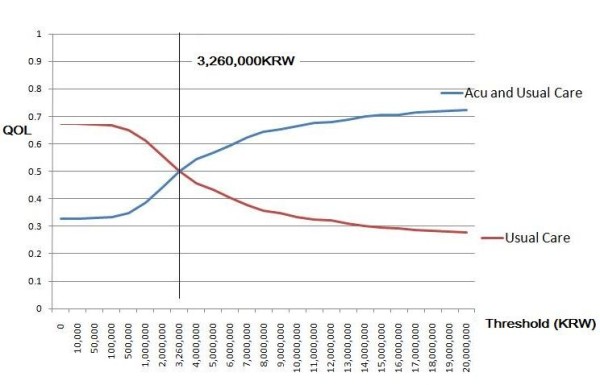
**CEAC for chronic low back pain (probabilistic sensitivity analysis)**.

In the probabilistic sensitivity analysis, all parameters were varied simultaneously in the ranges shown in Table 3. Random draws from each parameter distribution were performed; then the cost-effectiveness of each strategy was calculated. The procedure was repeated 10,000 times and we compared net monetary benefits of both alternatives at the range of 0 to 20,000,000 KRW of willingness to pay thresholds. Figure 5 displays the results of the analyses in the form of a cost-effectiveness acceptability curve (CEAC). This figure shows that if the threshold is over 3,260,000 KRW, the cost-effectiveness probability of collaborative treatment is higher than that of usual care. Furthermore, when the threshold was 20,000,000 KRW per QALY, the probability of preferring collaborative treatment was 72.3%, which is higher than usual care (26.3%).

### Value of information analysis

**Figure 6:**
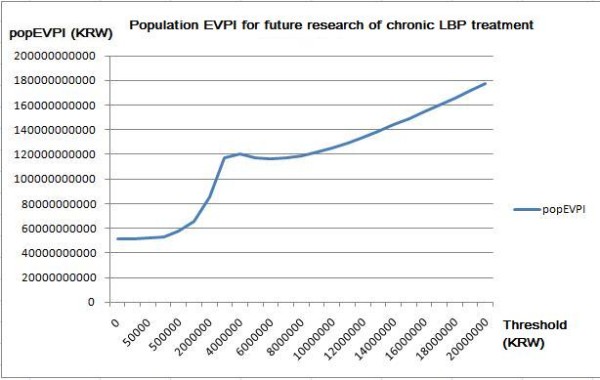
**Population EVPI analysis result**.

The value of information analysis (VOIA) results are displayed in Figure 6 as the maximum acceptable cost per research subject, which is calculated by multiplying the expected value of perfect information (EVPI) by the expected population.

The population EVPI was highest when the national threshold was 4,000,000 KRW, between 0 and 6,000,000 KRW. If the threshold increased to over 8,000,000 KRW, then the value of future research would exceed 120,000,000,000 KRW. According to this result, it would be reasonable to fund future research that evaluates the cost-effectiveness of collaborative treatment of acupuncture and usual care.

## Discussion

Recently, an increasing number of clinical research concerning acupuncture for LBP has been conducted in various countries [5,25,29-35]. In these papers, the results do not provide firm conclusions about the effectiveness of acupuncture for acute LBP. However, for chronic LBP patients, acupuncture was assumed to be effective for pain relief and functional improvement [15-17,36]. Furland et al. in their systematic review, concluded that acupuncture may be a useful adjunct to other therapies for chronic LBP [15]. However, reimbursement agents such as governments and insurers have recently required evidence of economic benefit along with clinical benefits to cover the treatments.

In 2010, the South Korean MOHW introduced several laws regarding medical provider employment and collaboration, which allow medical doctors and Oriental medical doctors (acupuncture qualified) to work together in the same facility and offer simultaneous collaborative treatment for the same disease. Previously, these types of collaborations were indirectly regulated by the limited reimbursing regulation of HIRA. These regulation changes will increase the frequency of collaboration between medical doctors and Oriental medical doctors; however, whether the national health insurance will cover this system is yet to be determined.

Therefore, we conducted this study to evaluate a collaboration of acupuncture and usual care for chronic LBP patients in the newly developing medical environments of South Korea.

Prior studies on this subject using patient level data have been published in other countries. In the UK, Ratcliffe and colleagues [6] conducted a pragmatic randomised clinical trial (RCT) and examined the cost-effectiveness of the addition of acupuncture treatment compared to usual care alone. They calculated the ICER of acupuncture at 24 months as £4,241 per QALY (1£ = 1,944.16 KRW). They concluded that assuming an implicit threshold of a maximum of willingness to pay of £20,000 per QALY, collaboration offers a modest health benefit for a minor extra cost to the National Health Service (NHS).

Witt et al. [5] also published pragmatic RCT results in Germany. In their study, they employed three arms of a mixed model, two arms of randomised groups, and one observational group, which were utilised to avoid the selection bias of participant inclusion. If the participants with severe LBP did not enter into the study and attempt to be treated by most available treatments, then the results would be exposed to a selection bias. Therefore, in the analysis, the researchers were able to examine the selection bias by comparing characteristics of randomised and non-randomised groups. Their results showed that the ICER of acupuncture was €10,526 per QALY (1€ = 1,742.12 KRW), and they concluded that acupuncture collaboration was relatively cost-effective at the threshold of €50,000.

These cost-effectiveness results, which are thought to be based on different medical institutions and economic conditions, could not be extrapolated to other countries. Hutubessy et al addressed that the simple extrapolation would be easy and quick, but it would result in misleading answers and could encourage inefficient decisions [37].

While the two studies mentioned above conducted the analyses using patient-level data from pragmatic RCTs, we used a Markov model simulation to obtain the discounted QALYs as a measure of effectiveness. The Markov model format allowed us to evaluate the economic impact of both alternatives over a five-year time horizon. The state definitions of chronic LBP in previous clinical trials were somewhat varying and confusing according to the purpose of each research. Therefore, we defined chronic LBP as persistent pain for 12 weeks or more, based on the clinical practice guidelines published by the Agency for Health Care Policy and Research (AHCPR) [38] and the Questionnaire of KNHNS 2007 [3].

The definition of 'usual care' could also vary based on each country's medical system. Although 54 clinical practice guidelines developed from South Korean medical system were listed in the official database, the specific guidelines for chronic LBP had not been established [39]. Therefore, we developed the questionnaire of usual care intervention lists from the ACP&APS' pharmacologic and non-pharmacologic interventions which are registered in the international practice guideline database [40]. Then we asked orthopaedic and rehabilitation specialists from a general hospital to identify the commonly using procedures in South Korea. Based on the survey results, we defined NSAIDs, heat therapy, electrotherapy and lumbar traction as the 'usual care' in South Korea.

The effectiveness of additive acupuncture treatment compared with usual care was derived from the improvement of state QOL of chronic LBP and changed transition probability to the Well state. The state QOL of chronic LBP treated with both alternatives was derived from the Witt et al. study results [5], and the difference of transition probabilities to the Well state was assumed from meta-analysis.

Although the effects come at a high cost, resulting in a marginal cost effectiveness ratio of nearly 3,421,394 KRW per QALY, the costs are less than the generally accepted societal threshold for willingness to pay at 20,000,000 KRW per QALY. In the probabilistic sensitivity analysis, there was a 72.3% chance that collaborative treatment would be cost-effective at a willingness to pay threshold of 20,000,000 KRW per QALY. This result indicates that for chronic LBP disease, acupuncture collaborative treatment could be acceptable to the National Health Insurance reimbursement lists.

Several limitations of the present analysis should be considered when interpreting its results. First, we could not include all available alternatives due to a lack of evidence. Despite the fact that herbal medicines, cupping, and other treatments are commonly used as alternative treatments in South Korea, we could not find appropriate papers that analysed the effectiveness of these alternatives. However, Weinstein et al. wrote that the ability of the model to make accurate predictions of future events is valuable, but not absolutely essential [13]. Because future events convey information that is not available at the time the model is developed, a model should not be criticised for failing to predict the future. Therefore, if these or other interventions establish evidences of their own effectiveness, we will take this new evidence into account for future analyses.

Second, the uncertainties of parameters in the Markov model could not be ruled out. Although, we examined the validation with a calibration, the uncertainty could not be solved perfectly. If future epidemiologic studies of the Korean population are published, then we could develop our model with more precision.

Third, when constructing the model of disease progression, the 'memoryless problem' of the Markov assumption could not be solved. When developing the model, the severity of disease that could differ in accordance with the disease progression should be considered using tunnel states. In addition, as mentioned in the Grotel et al. study, psychological, social and economic factors that differ among other countries could affect the chronification of LBP should also be considered in the modelling [21]. However, we could not find any appropriate data for building the tunnel state in the model.

Fourth, we could not avoid the discrepancy of evidence levels. In the cases of direct non-medical costs and usual care intervention lists, we had to depend on the low-level evidence of specialists' responses and simple hospital cost data.

Fifth, the heterogeneity of the data may affect the ability to generalise our findings. We used other countries' data for the meta-analysis, which could be a caveat to the full interpretation of effectiveness.

Despite these limitations, we built the Markov model of chronic LBP and conducted a cost-effectiveness analysis of usual care and acupuncture collaboration according to the reference case analysis methods. Finally, this study may offer evidence of allocative efficiency concerning the collaborative treatment of chronic LBP in the 2010 Korean medical environment.

## Conclusions

It is important to provide decision-makers with relevant information to help them determine if this new collaborative treatment strategy should be included in the National Health Insurance reimbursement list. The deterministic and sensitivity analyses results showed that collaborative treatment would be more cost-effective than usual care alone. Future research is needed to investigate details using domestic data, which could be reasonable based on the VOIA results.

## Abbreviations

ACP&APS: American College of Physicians and the American Pain Society; AHCPR: Agency for Health Care Policy and Rehabilitation; CEAC: Cost-effectiveness acceptability curve; EVPI: Expected value of perfect information; GDP: Gross domestic product; HIRA: Health Insurance Review and Assessment service; ICER: Incremental cost-effectiveness ratio; KNHNS: Korean National Health and Nutrition Survey; KRW: Korean rate Won; LBP: Low back pain; MOHW: Ministry of Health, Welfare and Family Affairs; NHS: National Health Service; QALY: Quality adjusted life year; QOL: Quality of life; RCT: Randomised clinical trial; VOIA: Value of information analysis.

## Competing interests

The authors declare that they have no competing interests.

## Authors' contributions

NKK was responsible for developing the research and drafting the manuscript. NKK originated the idea for this study and contributed to all phases of research and writing. In addition, he analysed and interpreted the data for study. TJL, BMY, and SMK participated in the analysis and interpretation of data, in the critical revision of the manuscript for important intellectual content, and in the study supervision. All authors reviewed and approved the final version of this manuscript.

## Appendices

The Appendices are available in Additional File 1.

## Pre-publication history

The pre-publication history for this paper can be accessed here:

http://www.biomedcentral.com/1472-6882/10/74/prepub

## Supplementary Material

Additional file 1 Systematic review protocol and Papers included in the systematic review Click here for file
