# Supplementary material for: An economic analysis of usual care and acupuncture collaborative treatment on chronic low back pain: A Markov model decision analysis
Source: BMC Complement Altern Med. 2010 Nov 25;10:74. doi: 10.1186/1472-6882-10-74 (PMC3009613; doi:10.1186/1472-6882-10-74)
Supplement: Additional file 1 — Systematic review protocol and Papers included in the systematic review [file 1472-6882-10-74-S1.DOC]

# Appendices

### Appendix 1 Systematic review protocol

The objectives are to ascertain the effectiveness of acupuncture for chronic LBP patients, and to draw out the relative effect of acupuncture collaborative treatment compared to usual care. Two independent authors conducted the reviews during the study. The search engines used were Ovid and Cochrane register of controlled trial (CENTRAL); domestic engines used were RISS and KOMS (Korean Oriental Medical Society) paper-searching databases. We considered medical technology development and public health environmental changes and thereby defined the search period as January 1999 through August 2009. We found randomised controlled trials (RCTs), pragmatic RCTs, systematic review studies of the subject, and RCTs evaluated with modified Jadad scores. Among 16 included studies, three studies were available to analyse transition probabilities to the Well state using head-to-head data of both alternatives. The rest of the studies were used to ascertain the acupuncture effects and treatment protocols.

### Appendix 2 Papers included in the systematic review

| **Paper** | **Type** |
| --- | --- |
| Furlan Andrea, D., W. van Tulder Maurits, et al., “Acupuncture and dry-needling for low back pain. Cochrane Database of Systematic Reviews.” DOI: 10.1002/14651858.CD001351.pub2, 2005. | Systematic Review & Meta-Analysis |
| Meng, C. F., D. Wang, et al., “Acupuncture for chronic low back pain in older patients: a randomized, controlled trial.” Rheumatology (Oxford, England) 1508-17, 2003. | RCT |
| Carlsson, C. P. and B. H. Sjölund, “Acupuncture for chronic low back pain: a randomized placebo-controlled study with long-term follow-up.” The Clinical journal of pain 296-305 | RCT |
| Brinkhaus, B., C. M. Witt, et al., Acupuncture in patients with chronic low back pain: a randomized controlled trial.“ Archives of internal medicine 450-7, 2006. | RCT |
| Kerr, D. P., D. M. Walsh, et al., “Acupuncture in the management of chronic low back pain: a blinded randomized controlled trial.” The Clinical journal of pain 364-70, 2003. | RCT |
| Leibing, E., U. Leonhardt, et al., “Acupuncture treatment of chronic low-back pain - a randomized, blinded, placebo-controlled trial with 9-month follow-up.” Pain 189-96, 2002. | RCT |
| Molsberger, A. F., J. Mau, et al., “Does acupuncture improve the orthopedic management of chronic low back pain-a randomized, blinded, controlled trial with 3 months follow up.” Pain 579-87, 2002. | RCT |
| Haake, M., H. H. Muller, et al., “German Acupuncture Trials (GERAC) for chronic low back pain: randomized, multicenter, blinded, parallel-group trial with 3 groups.” Arch Intern Med. 22;167(19):2072. 2007. | RCT |
| Thomas, K. J., H. MacPherson, et al., “Longer term clinical and economic benefits of offering acupuncture care to patients with chronic low back pain.” Health technology assessment (Winchester, England) iii-iv, ix-x, 1-109, 2005 | Pragmatic RCT |
| Witt, C. M., S. Jena, et al., “Pragmatic randomized trial evaluating the clinical and economic effectiveness of acupuncture for chronic low back pain. American journal of epidemiology.” 487-96, 2006. | Pragmatic RCT |
| Thomas, K. J., H. MacPherson, et al., “Randomised controlled trial of a short course of traditional acupuncture compared with usual care for persistent non-specific low back pain. BMJ (Clinical research ed.) 623, 2006. | Health Technology Assessment |
| Yuan, J., N. Purepong, et al., "Effectiveness of acupuncture for low back pain: a systematic review." Spine (Phila Pa 1976) 33(23): E887-900, 2008. | Systematic Review & Meta-Analysis |
| Manheimer, E., A. White, et al., “Meta-analysis: acupuncture for low back pain (Structured abstract).” Annals of Internal Medicine 651-663, 2005. | Systematic Review & Meta-Analysis |
| Kong, J. C., M. S. Lee, et al., "Randomized clinical trials on acupuncture in korean literature: a systematic review." Evid Based Complement Alternat Med 6(1): 41-8, 2009. | Systematic Review |
| Kwon et al., "The Short-term efficacy of acupuncture for chronic low back pain, randomized sham controlled trial." J Oriental Rehab Med 17(2):123-132 2007. | RCT |
